# Interrupted reprogramming of alveolar type II cells induces progenitor-like cells that ameliorate pulmonary fibrosis

**DOI:** 10.1038/s41536-018-0052-5

**Published:** 2018-09-04

**Authors:** Li Guo, Golnaz Karoubi, Pascal Duchesneau, Fabio Gava Aoki, Maria V. Shutova, Ian Rogers, Andras Nagy, Thomas K. Waddell

**Affiliations:** 1 Division of Thoracic Surgery, Toronto General Hospital Research Institute, University Health Network, University of Toronto, Toronto, ON Canada; 2 Lunenfeld Tanenbaum Research Institute, Sinai Health System, Toronto, ON Canada; 3 Department of Physiology, University of Toronto, Toronto, ON Canada; 4 Department of Obstetrics & Gynecology, University of Toronto, Toronto, ON Canada; 5 Institute of Medical Science, University of Toronto, Toronto, ON Canada; 6 Monash University, Melbourne, VIC Australia

## Abstract

We describe here an interrupted reprogramming strategy to generate “induced progenitor-like (iPL) cells” from alveolar epithelial type II (AEC-II) cells. A carefully defined period of transient expression of reprogramming factors (Oct4, Sox2, Klf4, and c-Myc (OSKM)) is able to rescue the limited in vitro clonogenic capacity of AEC-II cells, potentially by activation of a bipotential progenitor-like state. Importantly, our results demonstrate that interrupted reprogramming results in controlled expansion of cell numbers yet preservation of the differentiation pathway to the alveolar epithelial lineage. When transplanted to the injured lungs, AEC-II-iPL cells are retained in the lung and ameliorate bleomycin-induced pulmonary fibrosis. Interrupted reprogramming can be used as an alternative approach to produce highly specified functional therapeutic cell populations and may lead to significant advances in regenerative medicine.

**Electronic supplementary material:**

**Supplementary information** accompanies the paper on the *npj Regenerative Medicine* website (10.1038/s41536-018-0052-5).

## Pulmonary fibrosis: “Interrupted” reprogramming of lung cells shows therapeutic promise in mice

A modified reprogramming strategy helps expand populations of surfactant-producing lung cells in a dish without altering their cellular function. A team led by Thomas Waddell and Andras Nagy from the University of Toronto, Canada isolated alveolar type II cells from the lungs of mice. They transiently induced expression of four reprogramming factors in these cells for a defined period of time. Before this “interrupted” reprogramming, the lung cells had limited ability to continue replicating themselves. Afterwards, the cells could expand their numbers dramatically without entering a pluripotent state. Rather, the cells maintained their original function while also expressing genes associated with lung precursor cells, which could explain their proliferative ability. The cells, when transplanted into the injured lungs, helped ameliorate pulmonary fibrosis in a mouse model, suggesting that a similar cell-based therapy may be useful in people.

**Electronic supplementary material:**

**Supplementary information** accompanies the paper on the *npj Regenerative Medicine* website (10.1038/s41536-018-0052-5).

## Introduction

In the normal adult lung, the alveolar epithelium is composed of two major cell types, the alveolar epithelial type I (AEC-I) and alveolar epithelial type II (AEC-II) cells. It is generally thought that the squamous AEC-I cells are terminally differentiated cells that interface with pulmonary capillaries and cover >90% of the alveolar surface where the exchange of CO_2_/O_2_ takes place.^1^ In contrast, AEC-II cells are small cuboidal cells located in the corners of alveoli that cover only 5% of the alveolar surface. They are multifunctional cells that produce, secrete, and recycle pulmonary surfactants; regulate alveolar fluid balance; and synthesize and secrete a number of immune-modulatory proteins involved in host defense.^2^ Importantly, a subset of surfactant protein C-positive (SPC^+^) AEC-II cells serve as regional progenitors in the alveoli and differentiate into AEC-I cells, playing a crucial role in replenishing the alveolar epithelial barrier during both homeostasis and repair after injury.^3–5^

Impaired regeneration of injured alveolar epithelium has been observed in fibrotic interstitial lung diseases, including idiopathic pulmonary fibrosis (IPF). IPF is an irreversible, fatal interstitial lung disease with death occurring in most patients within 5 years of diagnosis. While not completely understood, increasing evidence suggests that the pathogenesis of IPF may be driven by alveolar epithelial cell dysfunction, followed by aberrant regeneration of epithelium, persistent activation of fibroblasts, and alterations in epithelial–mesenchymal communication with the extracellular matrix (ECM), together resulting in disruption of architecture and progressive loss of lung function.^6–8^ Currently, medical therapy for IPF is limited and lung transplantation is the only option for patients with end-stage IPF.^9,10^

A growing body of evidence describes putative progenitor cell populations in the distal lung that function to replenish or repair damaged epithelium.^5,11–16^ However, these cells are rare, which limits their expansion, and they usually change rapidly upon in vitro culture.^17–20^ Importantly, in several disease or injury states, endogenous progenitors are limited in number and function.^21^ Thus recent focus has been placed on using cell-based therapeutic approaches for ameliorating fibrosis via a cell replacement strategy. Tremendous efforts have been made in application of bone marrow cells (BMCs),^22–24^ mesenchymal stromal cells (MSCs)^25–28^ and respiratory epithelial cells differentiated from pluripotent sources such as embryonic stem and induced pluripotent stem cells (ESCs and iPSCs, respectively).^29–32^ Among these, MSCs have advantages as a practical source for use in cell-based therapies for lung disease. The vast majority of studies report some biological effects after MSC delivery during the early inflammatory phase of bleomycin (BLM)-induced pulmonary fibrosis. However, low levels of cell engraftment or retention suggest paracrine-based mechanisms of action responsible for repair.^27,33,34^ In contrast, freshly isolated AEC-II cells appear to be effective even after administration in later stages of IPF where fibrosis is prevalent.^35,36^ However, the practical usages of freshly isolated AEC-II cells are limited by donor availability and maintenance in culture.^19,20^ Despite recent progress in obtaining distal epithelial cells from directed differentiation of ESC and iPSCs,^29–32^ protocols remain limited by yield and purity of AEC-II cells. Furthermore, the pluripotent nature of ESC and iPSCs still present a potential risk of tumorigenicity, which must be addressed for clinical applicability.^37,38^ Regardless of cell source, for most cell therapy applications, the cells will need externally controllable proliferative capacity to maintain homeostasis or respond to injury.

We present an interrupted reprogramming strategy that provides an alternative approach to generate a functional AEC-II population with high purity. We took advantage of the rapid induction of cell proliferation and residual epigenetic “memory” retained during the early phase of reprogramming^39–46^ to create cells we have termed “induced progenitor-like (iPL) cells.” We achieved this by optimizing and carefully controlling the duration of transient expression of iPSC reprogramming factors (Oct4, Sox2, Klf4, and c-Myc (OSKM)), turning off their expression prior to reaching independent pluripotency. Interrupted reprogramming allows controlled expansion yet preservation of AEC-II lineage commitment and rescues the limited in vitro clonogenic capacity of AEC-II cells. Importantly, iPL cells derived from AEC-II cells ameliorate BLM-induced pulmonary fibrosis in vivo. The ability to produce highly specified therapeutic cell populations retaining critical functions may have significant implications for cell-based therapies.

## Results

### Interrupted reprogramming rescues the limited clonogenic capacity of AEC-II cells while achieving expansion

**Fig. 1:**
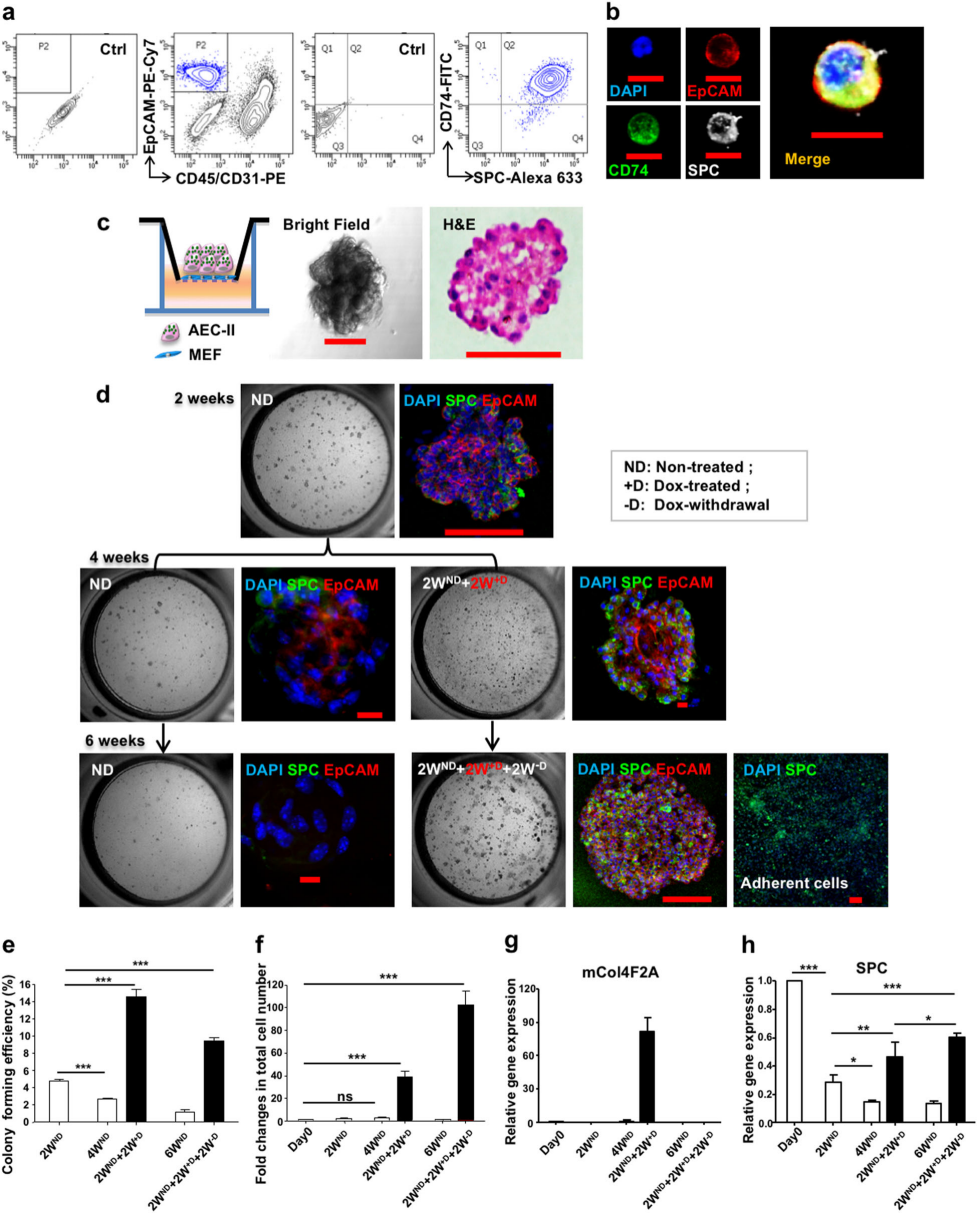
Interrupted reprogramming rescues the in vitro limited clonogenic capacity of AEC-II cells while achieving expansion in cell numbers. **a** Flow cytometric analysis of freshly isolated lung cells using a modified elastase-based protocol, showing epithelial cells marked with antibodies specific for SPC and CD74. Over 95% of EpCAM^+^, CD45^−^, CD31^−^ cells are AEC-II cells co-expressing SPC and CD74. **b** Freshly isolated AEC-II cells express EpCAM (red), CD74 (green), and SPC (gray); nuclei are stained with DAPI (blue). **c** Isolated AEC-II cells are able to give rise to colonies in a Matrigel-based 3D culture system. Light microscopy bright-field images and H&E staining are shown. **d** The left panel shows bright-field images depicting the generation of colonies in the absence (ND), presence of Dox (+D), and upon subsequent Dox-withdrawal (−D). The right panel shows confocal microscopic images of colonies obtained from each condition, showing nuclear stain DAPI (blue), SPC (green), and EpCAM (red). The colony-forming efficiency (**e**) and the fold changes in total cell number (**f**) shown relative to day 0 seeded cells (5000 cells/well). Expression levels of **g** mCol4F2A and **h** SPC in cells obtained from each condition, as measured by qRT-PCR comparing fold differences in the expression in day 0 freshly isolated AEC-II cells. In **a**, data are representative of a minimum of three biological replicates. For **e**–**h**, values are mean ± S.D. of three independent biological replicates. *p < 0.05; **p < 0.001; ***p < 0.0001. Scale bar, 10 µm (**b**, **d**); 50 µm (**c**—H&E); 100 µm (**c**—bright field and **d**)

AEC-II cells play a central role in alveolar epithelial repair and regeneration. In vitro, although AEC-II cells can give rise to alveolar-like colonies, they possess limited clonogenic capacity that decreases with passaging.^47^ Importantly, their number and function decline with age and in certain pathological conditions,^48,49^ including IPF.^50^ To determine whether interrupted reprogramming is able to rescue the in vitro limited clonogenic capacity of AEC-II cells, we isolated AEC-II cells from R26-rtTA/Col1a1::tetO-4F2A double transgenic mice^51^ enabling expression of OSKM following treatment with doxycycline (Dox). AEC-II cells were isolated using a modified elastase-based protocol and characterized using anti-CD74^52^ in addition to the classical AEC-II marker anti-SPC. Flow cytometric analysis of freshly isolated cells showed that >95% of CD45^neg^CD31^neg^EpCAM^pos^ AEC-II cells expressed both markers (Fig. 1a, b). Isolated AEC-II cells gave rise to colonies when cultured in a Matrigel-based three-dimensional culture system (Fig. 1c). Consistent with previous reports,^47^ there was a gradual reduction in colony-forming efficiency (CFU%) with passaging (Fig. 1d—ND groups). We optimized iPL cell induction conditions for AEC-II cells and found an initial 2 weeks of culture without Dox (ND) prior to Dox treatment (+D) (named “late induction”) appeared advantageous. Induction of AEC-II cells (2W^ND^ + 2W^+D^) significantly increased the CFU% and total number of cells (Fig. 1d–f). Subsequent withdrawal of Dox for 2 weeks (2W^ND^ + 2W^+D^ + 2W^−D^) resulted in a decrease in transgene OSKM (mCol4F2A) expression (Fig. 1g) and a decrease in CFU%. However, the colonies that did develop were larger and numerous adherent epithelial-like cells were observed, likely contributing to the increased total number of cells obtained (Fig. 1d, e). Overall, this resulted in a 100-fold cell expansion (Fig. 1f). Non-treated AEC-II cells gradually lost their phenotype with passaging, possibly due to the differentiation to AEC-I-like cells. In contrast, the AEC-II phenotype was well preserved in the 2W^ND^ + 2W^+D^ induced cells, even after withdrawal of Dox for 2 weeks (2W^ND^ + 2W^+D^ + 2W^−^^D^) (Fig. 1d, h). This data showed that interrupted reprogramming of AEC-II is able to rescue the limited passaging capacity of AEC-II colonies and efficiently expand cells while preserving AEC-II lineage commitment.

### Interrupted reprogramming allows preservation of AEC-II lineage commitment without traversing the pluripotent state

**Fig. 2:**
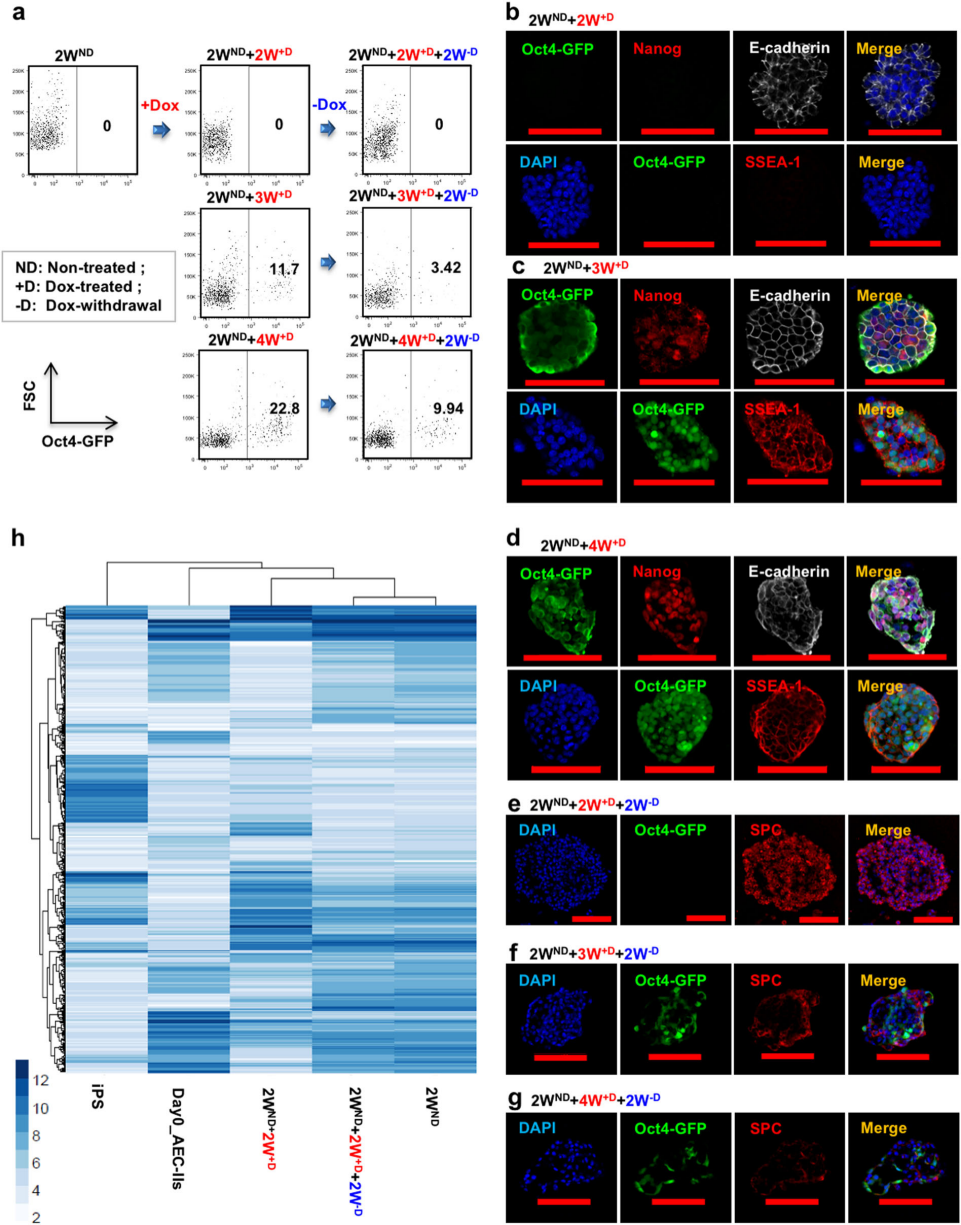
A defined period of interrupted reprogramming allows preservation of AEC-II phenotype without traversing pluripotency. **a** Flow cytometric analysis of endogenous Oct4-GFP expression in passaged cultured AEC-II cells (2W^ND^) derived from R26-rtTA/Col1a1::tetO-4F2A;Oct4-GFP mice under Dox induction (+D) and subsequent Dox withdrawal (−D) showed ≥3 weeks of induction resulted in factor-independent Oct4-GFP expression. Confocal microscopic images depict the expression of pluripotency markers in cells treated with Dox for **b** 2 weeks (2W^ND^ + 2W^+D^), **c** 3 weeks (2W^ND^ + 3W^+D^), and **d** 4 weeks (2W^ND^ + 4W^+D^), with nuclear stain DAPI (blue), Oct4-GFP (green), Nanog/SSEA-1 (red), and E-cadherin (gray). Confocal microscopic images show preservation of the AEC-II phenotype in Dox-treated cells with subsequent 2-week culture in Dox-free media, **e** 2W^ND^ + 2W^+D^ + 2W^−D^, **f** 2W^ND^ + 3W^+D^ + 2W^−D^, and **g** 2W^ND^ + 4W^+D^ + 2W^−D^, with nuclear stain DAPI (blue), Oct4-GFP (green), and SPC (red). **h** Unbiased clustering analysis of all genes expressing ≥2-fold change. In **a**, data are representative of a minimum of three independent biological replicates. Scale bar, 50 µm (**c**—bottom panel and **d**—top panel); 100 µm (**b**, **c**—top panel, **d**—bottom panel, and **e**–**g**)

To evaluate the extent of reprogramming of the cells, we cultured AEC-II cells derived from R26-rtTA/Col1a1::tetO-4F2A;Oct4-GFP mice^53^ in which the activation of endogenous Oct4 could be monitored by green fluorescent protein (GFP) expression. Flow cytometric analysis represented Oct4-GFP expression in the overall bulk population under different conditions, while immunostaining panels are focused on a few selected Oct-GFP^+^ colonies for detection of the activation of other pluripotency markers and SPC expression. AEC-II cells treated with Dox for ≥3 weeks resulted in the presence of Dox-independent Oct4-GFP cells (Fig. 2a) expressing the pluripotency markers: Nanog and SSEA-1 (Fig. 2c, d). In contrast, AEC-II cells cultured in the presence of Dox for only 2 weeks (2W^ND^ + 2W^+D^) cells did not contain any Oct4-GFP^+^ cells and showed no expression of other pluripotency markers (Fig. 2a, b). We assessed the ability of induced AEC-II cells to return to their original AEC-Il phenotype following withdrawal of Dox. Withdrawal of Dox after a 2-week induction (2W^ND^ + 2W^+D^ + 2W^−D^) resulted in Oct4-GFP^−^SPC^+^ colonies showing the return to the AEC-II phenotype (Fig. 2e). In contrast, colonies obtained upon Dox withdrawal after ≥3 weeks' induction (2W^ND^ + 3W^+D^ + 2W^−^^D^, 2W^ND^ + 4W^+D^ + 2W^−D^) contained Oct4-GFP cells and fewer SPC^+^ cells suggesting that subsets of ≥3 week induced cells had already passed the point of no return (Fig. 2f, g). The ability of these cells to return to the original phenotype was also evaluated using genome-wide transcriptional profiling (Fig. 2h). There were significant changes simply related to the adaptation to in vitro culture. Cells cultured for 2 weeks without Dox showed significant changes compared to freshly isolated AEC-II cells. Having said that, gene expression moved toward an iPS pattern during Dox induction but returned to closely resemble the gene expression of these “starting point” cells upon Dox withdrawal. Taken together, our results showed that, with our defined length of interrupted reprogramming, specifically this 4-week protocol (2W^ND^ + 2W^+D^) allows the greatest expansion of AEC-II cells without potentially traversing the pluripotent state.

### Interrupted reprogramming induces the expression of alveolar progenitor markers Hopx and α6β4

**Fig. 3:**
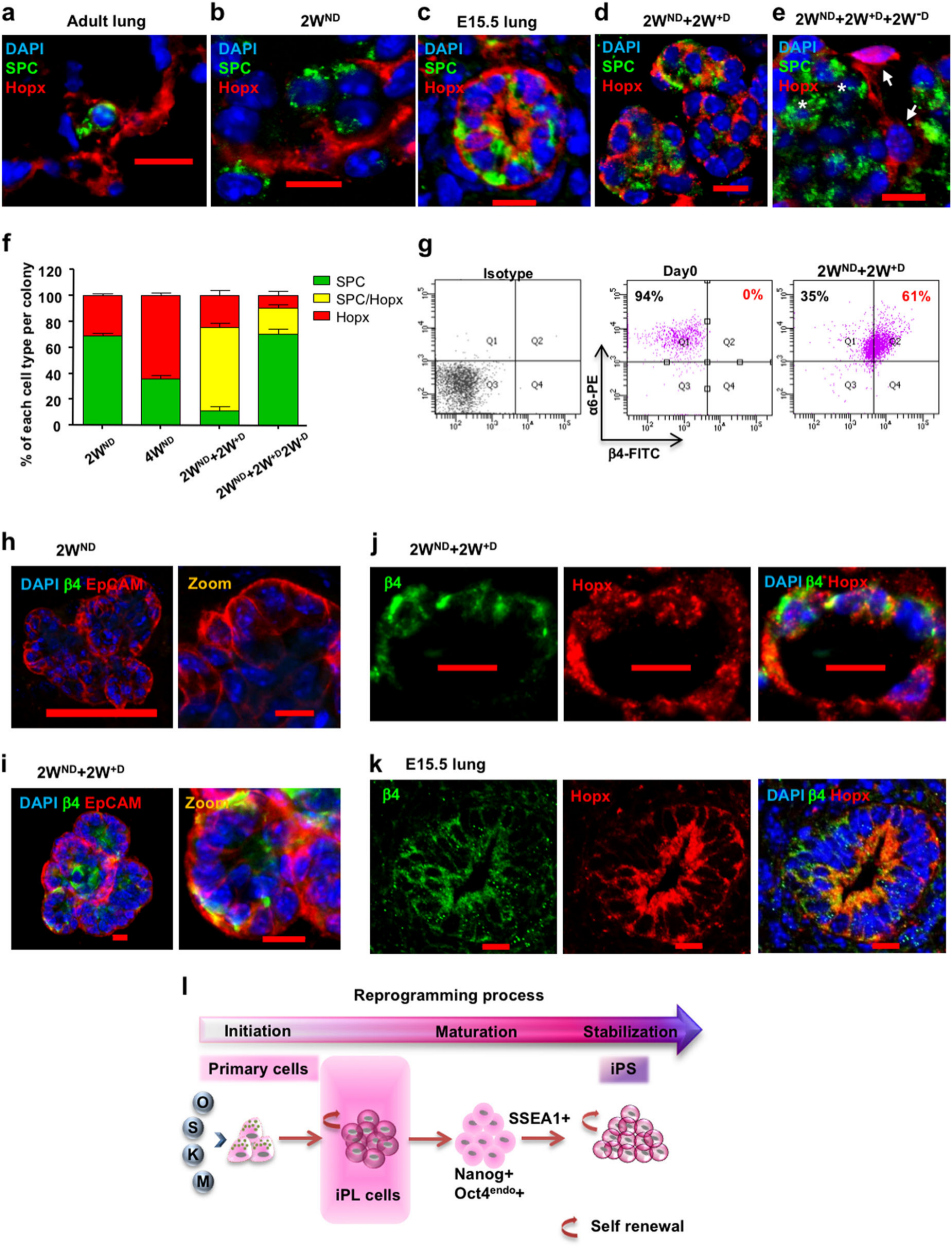
The expression of alveolar progenitor markers in AEC-II-iPL cells. Confocal microscopic images depict the expression of Hopx and SPC in: **a** adult lung; **b** colonies derived from AEC-II cells after a 2-week culture period in the absence of Dox (2W^ND^); **c** E15.5 lungs; **d** passaged 2W^ND^ colonies induced with Dox for 2 weeks (2W^ND^ + 2W^+D^); **e** colonies were passaged and cultured without Dox for 2 subsequent weeks (2W^ND^ + 2W^+D^ + 2W^−D^), with nuclear stain DAPI (blue), SPC (green), and Hopx (red). **f** Quantification of each cell type (SPC single positive; Hopx single positive; SPC and Hopx double positive) in colonies obtained from different groups. **g** Flow cytometric analysis of integrin α6 and β4 expression levels in day 0 freshly isolated AEC-II cells and 2W^ND^ + 2W^+D^ cells. Confocal microscopic images of colonies derived from AEC-II cells after 2-week culture without Dox (2W^ND^) **h** and 2W^ND^ + 2W^+D^ colonies (2W^ND^ + 2W^+D^) **i**, showing nuclear stain DAPI (blue), β4 (green), and EpCAM (red). Confocal microscopic images depicting the co-expression of Hopx and SPC in 2W^ND^ + 2W^+D^ cells **j** and E15.5 lungs **k**, with nuclear stain DAPI (blue), β4 (green), and Hopx (red). **l** Cartoon depicting the generation of iPL cells using interrupted reprogramming. For **f**, values are mean ± S.D. of three independent biological replicates. In **g**, data are representative of a minimum of three independent biological replicates. Scale bar, 10 µm (**a**–**e**, **h**—zoom, **i**–**k**), 100 µm (**h**—left panel)

Lineage tracing studies have demonstrated that both AEC-I and AEC-II cells arise from a bipotent progenitor cell during lung development, whereas AEC-I cells derive from subsets of AEC-II cells after birth and in adult lung. Hopx (homeodomain only protein x) first expressed in the embryonic lung at day 15.5 marks a bipotent alveolar progenitor in the developing lung.^54^ Hopx is turned off in maturing AEC-II cells and becomes restricted to AEC-I cells in the mature lung (Fig. 3a). We observed that alveolar-like colonies, generated by freshly isolated AEC-II cells, cultured in Matrigel conditions for 2 weeks without Dox (2W^ND^), contained SPC^+^ AEC-II cells as well as Hopx^+^ AEC-I cells. Notably, no co-expression of SPC and Hopx was observed (Fig. 3b). In contrast, the majority of AEC-II cells after interrupted reprogramming expressed SPC with a large proportion (85.5 ± 4.0% of SPC^+^ cells) co-expressing Hopx (Fig. 3d, f) with a pattern similar to that seen in E15.5 lungs (Fig. 3c, S1). An increased expression of SPC in these Hopx^+^ SPC^+^ cells could contribute to the overall SPC upregulation in the 2W^ND^ + 2W^+D^ bulk population compared to SPC expression in the 2W^ND^ population (Fig. 1h). Withdrawal of Dox (2W^ND^ + 2W^+D^ + 2W^−D^) resulted in a reduction in the number of SPC^+^/Hopx^+^ cells (Fig. 3e, f). While integrin α6 is ubiquitously expressed in lung epithelium,^47^ coexpression of integrin α6β4 marks a rare progenitor sub-population within normal distal lung involved in maintenance of AEC-II cells during lung repair.^14,16^ Flow cytometry showed β4 to be absent in the native α6^+^ AEC-II starting population but expressed in >60% of AEC-II cells after interrupted reprogramming (Fig. 3g). Immunostaining confirmed β4 expression (Fig. 3h, i) and showed that β4 was co-expressed with Hopx in these cells, similar to that seen in distal epithelium of E15.5 lungs (Fig. 3j, k). Collectively, our results suggest that interrupted reprogramming rescues the limited clonogenic capacity of AEC-II cells in vitro as a result of activation of an alveolar progenitor state. We have therefore termed these cells, AEC-II-derived iPL cells (AEC-II-iPL cells) (Fig. 3l).

### AEC-II-iPL cells ameliorate BLM-mediated pulmonary fibrosis

**Fig. 4:**
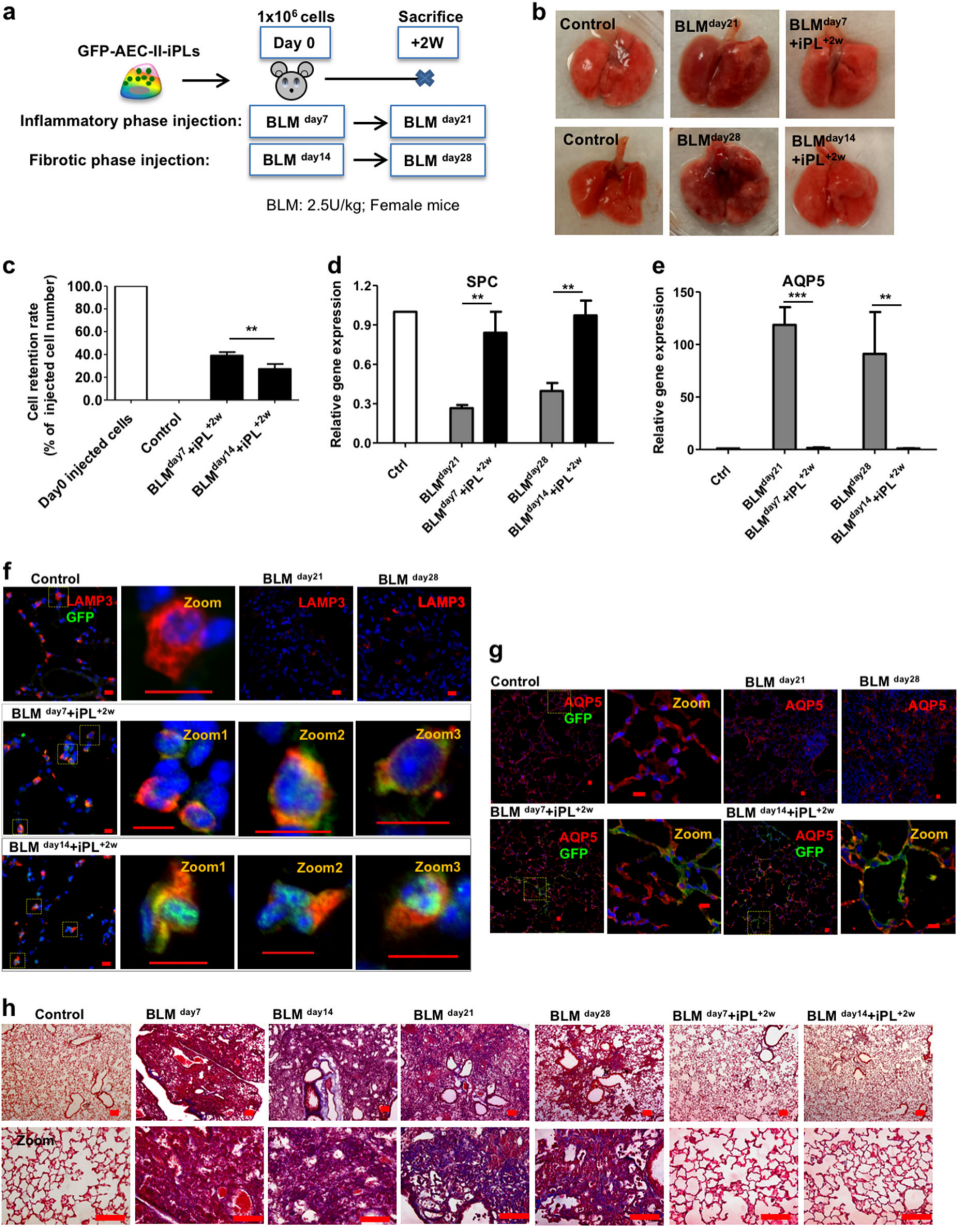
AEC-II-iPL cells are able to engraft and contribute to alveolar epithelial lineage in vivo. **a** The experimental schema of the in vivo study using female recipients is shown. **b** Representative images of whole lungs from all the experimental groups at days 21 and 28 after bleomycin (BLM) treatment. **c** Retention rate of the delivered GFP^+^ cells in the recipient lungs (percentage of day 0 injected cells) was calculated using genomic GFP measured at days 21 and 28 of BLM treatment (relative to β-actin GFP lungs) measured by PCR. Expression levels of **d** SPC and **e** AQP5 in the recipient lungs, as measured by qRT-PCR, comparing fold differences in the expression to control lungs. Confocal microscopic images of alveolar epithelium of the saline control, BLM untreated (BLM ^day21^, BLM ^day28^), and BLM cell-treated (BLM ^day7^ + iPL^+2W^, BLM ^day14^ + iPL^+2W^) animals showing nuclear stain DAPI (blue), GFP (green), and LAMP3 (red) **f** or AQP5 (red) **g**, respectively. **h** Representative images of Masson’s trichrome staining of all the experimental lungs after 7, 14, 21, and 28 days of BLM-induced pulmonary fibrosis showing interstitial collagen (blue) was greatly diminished in BLM transplanted female recipient lungs 2 weeks after receiving AEC-II-iPL cells at days 7 and 14 of BLM treatment (BLM ^day7^ + iPL^+2w^, BLM ^day14^ + iPL^+2w^). For **c**–**e**, values are mean ± S.D. of three independent biological replicates **p < 0.001; ***p < 0.0001. Scale bar, 100 µm (**f**–**h**); 10 µm (**f** and **g**—zoom)

BLM lung injury predominantly affects the alveolar compartment, has a prominent early inflammatory phase, and later results in fibrotic remodeling, which has some similarities to IPF in humans.^55^ Animal studies have demonstrated that MSCs reduce acute inflammation and protect against subsequent development of fibrosis in BLM-induced injury, only when cells were administered in the early inflammatory phase.^27^ Therefore, we examined the engraftment and therapeutic benefit of AEC-II-iPL cell treatment in ameliorating pulmonary fibrosis at both the inflammation and fibrotic phases of BLM-induced injury (Fig. 4a). Female recipients were sacrificed 2 weeks after cell delivery. Lungs of animals treated with AEC-II-iPL cells (with a constitutive GFP marker) either during the inflammatory (BLM ^day7^) or fibrotic phase (BLM ^day14^) showed a dramatically improved external appearance (Fig. 4b). Retention of AEC-II-iPL cells was greater when cells were administered during the inflammatory phase (39.1 ± 3.1% of the initial injected cell number) compared to cells delivered during the fibrotic phase (27.3 ± 4.4%) (Fig. 4c). SPC mRNA levels were also restored in AEC-II-iPL cell-treated lungs (Fig. 4d). BLM treatment results in aberrant differentiation of AEC-II cells into AEC-I cells due to the ECM abnormalities and interruption of the epithelial basement membrane, with abundant AQP5-expressing cells and upregulated expression of AQP5 mRNA. In AEC-II-iPL cell-treated lungs, AQP5 mRNA was expressed at a normal level (Fig. 4e). Compared to control lungs, there were few AEC-II cells (as marked by LAMP3^5^) following BLM treatment, whereas numerous LAMP3^+^ GFP^+^ cells were seen in the lungs treated with AEC-II-iPL cells (Fig. 4f). AQP5-expressing GFP cells were found, suggesting that the delivered AEC-II-iPL cells contributed directly to this lineage as well (Fig. 4g).

There was evidence of inflammation and epithelial damage, with infiltration of inflammatory cells in peribronchiolar and interstitial regions 7 days after BLM. Figure 4h shows Masson’s trichrome-stained lung sections. Hematoxylin–eosin staining is shown in Figure S2a. Two weeks later, without cell therapy, lungs showed extensive fibrosis and collagen deposition. In contrast, AEC-II-iPL cell-treated lungs showed fewer fibrotic lesions and less collagen in the parenchyma with normal alveolar architecture, suggesting that AEC-II-iPL cells can prevent the progression to fibrosis. In lungs treated with AEC-II-iPL cells 14 days after BLM administration (fibrotic phase), there was still a marked reduction in the number of α-SMA^+^ cells in the interstitium (Fig. S2b). In addition, we observed a significant reduction in the expression of mesenchymal cell-related genes in AEC-II-iPL cell-treated lungs (Fig. S2c, d). Taken together, AEC-II-iPL cells can engraft as epithelial cells and may be useful as a cell replacement therapy for ameliorating pulmonary fibrosis, even when administrated at the established fibrotic phase.

### AEC-II-iPL cells are able to engraft and differentiate to mature AEC-II and AEC-I cells in severely injured alveolar epithelium of male mice

**Fig. 5:**
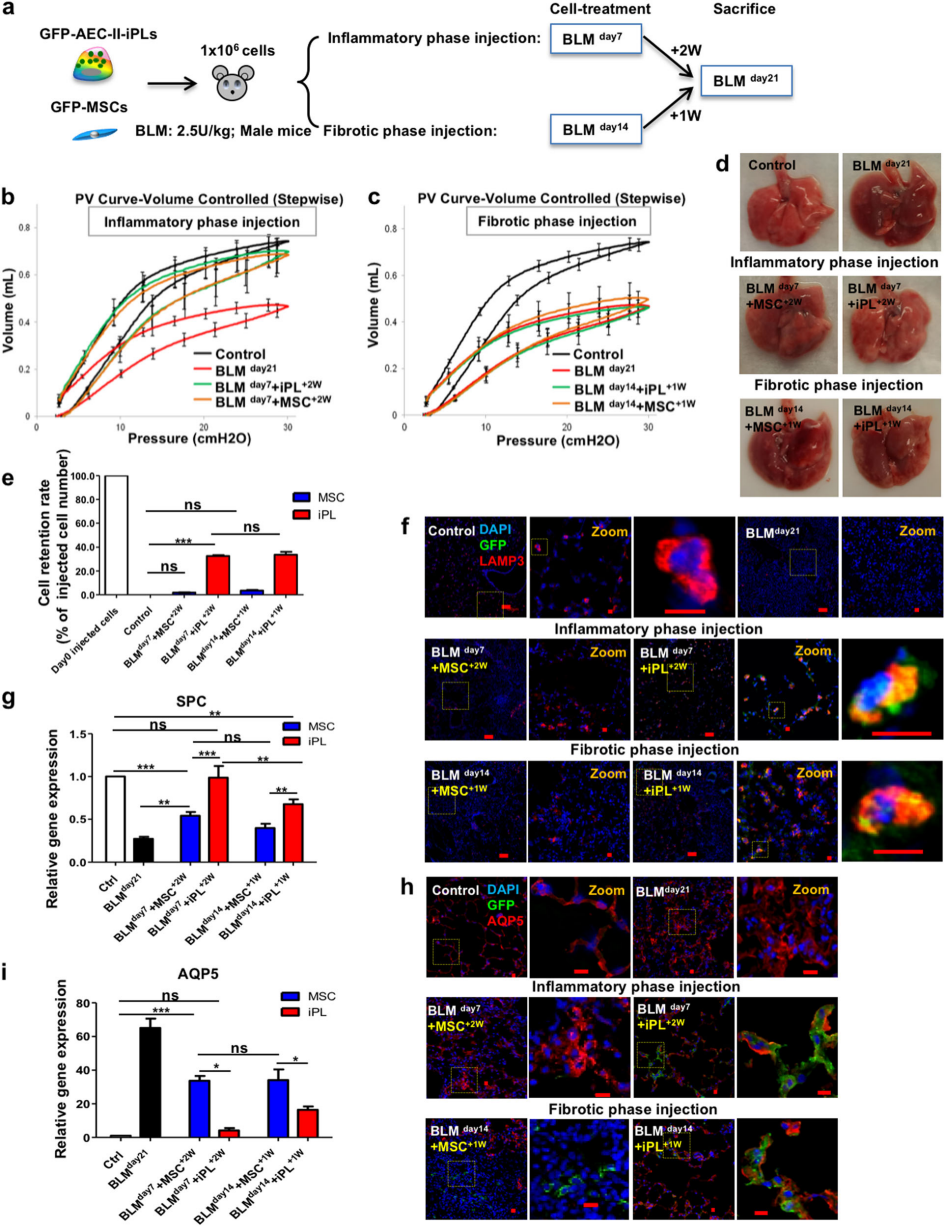
AEC-II-iPL cells are able to engraft and differentiate to mature AEC-II and AEC-I cells in severely fibrotic lungs. **a** Experimental schema depicting the in vivo study using male recipients. Lung mechanical function measured by FlexiVent showing PV curves of all the experimental groups, **b** BLM cell-treated male recipient lungs 2 weeks after receiving either AEC-II-iPL or MSC cells at day 7 of BLM treatment (BLM ^day7^ + iPL^+2W^, BLM ^day7^ + MSC^+2W^), and **c** BLM cell-treated male recipient lungs 1 week after receiving AEC-II-iPL cells or MSC at day 14 of BLM treatment (BLM ^day14^ + iPL^+1W^, BLM ^day14^ + MSC^+1W^). **d** Representative images of whole lungs from all the experimental groups at day 21 of bleomycin (BLM) treatment. **e** The cell retention rate of engrafted GFP cells in recipient lungs (percentage of day 0 injected cells) was calculated using genomic GFP at day 21 of BLM treatment (relative to β-actin GFP lungs) measured by PCR. **f** Confocal microscopic images of alveolar epithelium of the saline control, BLM untreated (BLM ^day21^), and BLM cell-treated at day 7 (BLM ^day7^ + iPL^+2W^, BLM ^day7^ + MSC^+2W^) or at day 14 (BLM ^day14^ + iPL^+1W^, BLM ^day14^ + MSC^+1W^) animals showing nuclear stain DAPI (blue) and LAMP3 (red) and GFP (green) expression. **g** Expression levels of SPC in recipient lungs, as measured by qRT-PCR, comparing fold differences in the expression to control lungs. **h** Confocal microscopic images of alveolar epithelium of the saline control, BLM untreated (BLM ^day21^), and BLM cell-treated at day 7 (BLM ^day7^ + iPL^+2W^, BLM ^day7^ + MSC^+2W^) and at day 14 (BLM ^day14^ + iPL^+1W^, BLM ^day14^ + MSC^+1W^) showing nuclear stain DAPI (blue) and AQP5 (red) and GFP (green). **i** Expression levels of AQP5 in the recipient lungs, as measured by qRT-PCR, comparing fold differences in the expression to control lungs. For **b**, **c**, **e**, **g**, **i**, values are mean ± S.D. of three independent biological replicates *p < 0.05; **p < 0.001; ***p < 0.0001. Scale bar, 100 µm (**f**); 10 µm (**f**—zoom and **h**)

Male sex is associated with a significant increase in the prevalence of fibrotic interstitial lung diseases, including IPF.^56,57^ In BLM model of pulmonary fibrosis, male mice develop more prominent fibrotic disease compared with female mice, and the pulmonary functional alterations can be detected by FlexiVent measurement.^58^ We therefore selected a male mouse BLM model to do a direct comparison of AEC-II-iPL cells with MSCs in improving lung function. As no significant pulmonary dysfunction could be detected 28 days after BLM compared to saline control (data not shown), we performed lung function analysis using a FlexiVent system 21 days after BLM. The engraftment and therapeutic benefit of AEC-II-iPL cells and MSCs were examined following cell delivery at both the inflammatory and fibrotic phases of BLM-induced injury. Animals were sacrificed at BLM ^day21^ (Fig. 5a). Pressure–volume (PV) loops were obtained using a quasi-static pressure-controlled PV ramp perturbation. Lungs treated with both AEC-II-iPL and MSC cells during the inflammatory phase (BLM ^day7^) showed improved lung function, closer to that of control lungs (Fig. 5b). No significant improvement of lung function was found in animals treated with either cell types during the fibrotic phase (Fig. 5c). This may due to short in situ period of delivered cells (injected on BLM ^day14^ and measurement performed on BLM ^day21^). Similarly, lungs of animals treated with AEC-II-iPL cells during the inflammatory (BLM ^day7^) phase showed an improved external appearance compared to other groups (Fig. 5d). Compared to the poor cell retention seen in MSC-treated lungs, AEC-II-iPL cells were retained in higher numbers when delivered during either the inflammatory or fibrotic phase (Fig. 5e). The contribution of engrafted cells to the alveolar epithelial lineage in recipient lungs was assessed by immunostaining. Compared to control lungs, there were few AEC-II cells (as marked by LAMP3) following BLM treatment, whereas numerous LAMP3^+^ GFP^+^ cells were seen in lungs treated with AEC-II-iPL cells at either the inflammatory or fibrotic phases. The LAMP^+^ cells found in MSC-treated lungs were not GFP^+^, suggesting that MSCs do not directly contribute to the AEC-II lineage (Fig. 5f). SPC mRNA levels were also greatly restored in AEC-II-iPL cell-treated lungs (Fig. 5g). AQP5-expressing GFP^+^ cells were found in AEC-II-iPL cell-treated lungs, whereas AQP5^+^ cells found in MSC-treated lungs were not GFP^+^ (Fig. 5h, i).

**Fig. 6:**
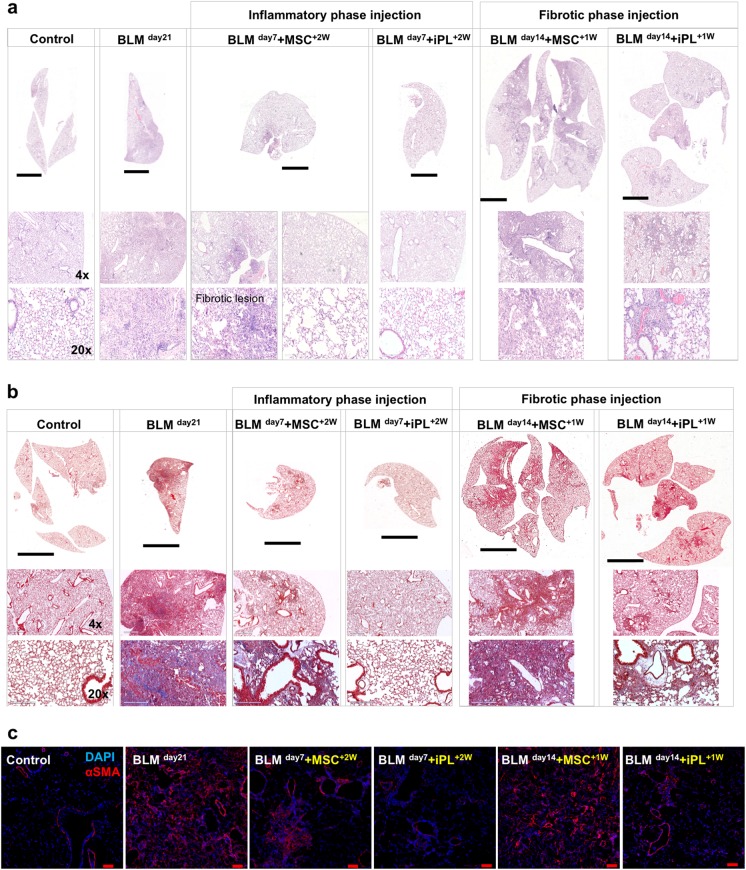
Treatment with AEC-II-iPL cells ameliorates severe pulmonary fibrosis. Representative images of lung histopathology are shown for all the experimental groups after 21 days of BLM-induced pulmonary fibrosis, the saline control, BLM untreated (BLM ^day21^), and BLM cell-treated at day 7 (BLM ^day7^ + iPL^+2W^, BLM ^day7^ + MSC^+2W^) or day 14 (BLM ^day14^ + iPL^+1W^, BLM ^day14^ + MSC^+1W^). **a** lung sections were stained with hematoxylin–eosin, **b** Masson’s trichrome staining of all the experimental lungs showing a remarkable decrease of interstitial collagen deposition (the blue stain) in the BLM-injured lungs treated with AEC-II-iPL cells compared with the ones treated with MSC. **c** Immunostaining of lung sections of all experimental groups with nuclear stain DAPI (blue) and αSMA (red). Scale bar, 3 mm (**a**—whole section tile scan), 4 mm (**b**—whole section tile scan); 600 µm (**a**, **b**—×4); 200 µm (**a**, **b**—×20) and 100 µm (**c**)

Hematoxylin–eosin (Fig. 6a) and Masson’s trichrome (Fig. 6b) staining showed evidence of severe inflammation, extensive fibrosis, and collagen deposition developed in the BLM-treated male lungs. In contrast, AEC-II-iPL cell-treated lungs showed fewer fibrotic lesions and less collagen in the parenchyma compared to the MSC-treated lungs, suggesting that AEC-II-iPL cells can reduce the progression to fibrosis. Compared to the lungs treated with MSC, AEC-II-iPL cell-treated lungs, when cells were administered during the fibrotic phase, showed a marked reduction in the number of α-SMA^+^ cells in the interstitium (Fig. 6c).

## Discussion

We show here that a carefully defined period of interrupted reprogramming, where the reprogramming process is initiated but not allowed to pass the point of no return,^59^ is able to rescue the limited in vitro clonogenic capacity of AEC-II cells. Importantly, our results demonstrate that using interrupted reprogramming we can achieve controlled expansion of an otherwise difficult to maintain AEC-II phenotype in vitro.^19,20,60^ AEC-II-iPL cells, when delivered to the injured lungs, are able to engraft and contribute to both the AEC II and AEC-I cell lineage. They ameliorated BLM-induced pulmonary fibrosis. Our result showed that interrupted reprogramming is not only able to achieve controlled expansion of AEC-II cells but also may result in the “de-differentiation” of the cells to a bipotent PL state. While it remains to be determined whether AEC-iPL cells truly recapitulate a distinct lung epithelial progenitor population, our data suggest at least a phenotypic likeness (SPC^+^ Hopx^+^ α6β4^+^) to an embryonic bipotent alveolar progenitor.^54^ Interrupted reprogramming may be useful as an alternative approach to generate large numbers of functional AEC-II cells with high purity.

There continues to be an interest in cell-based therapeutic approaches for lung diseases, including IPF.^23^ A variety of cell types have been investigated with the majority of studies focusing on MSCs isolated from bone marrow,^26,27^ umbilical cord,^61^ placenta,^28^ and adipose^25^ tissues. A limitation to using these cells types is they act mainly through paracrine signaling with little contribution to lung regeneration through engraftment and/or differentiation. Numerous animal studies have demonstrated that paracrine signaling by MSC reduces acute inflammation and protects against subsequent development of fibrosis in BLM-induced injury.^27,62,63^ However, these beneficial effects were only observed when cells were administered in the early inflammatory phase of lung injury but not in the later fibrotic phase^27^ where in fact there is even a possibility of stimulating detrimental fibroblast proliferation and ECM deposition.^64^ Nevertheless, a number of clinical studies using MSC are currently underway for IPF (NCT02013700, NCT02135380, NCT01919827, NCT01385644, NCT02277145).^23^ Low MSC retention suggests that the therapeutic effects are likely via a paracrine mechanism rather than engraftment. Unlike MSCs, we found that AEC-II-iPL cells are retained at a high rate, potentially even engraft, and appear to contribute directly to the alveolar epithelial lineage. AEC-II-iPL cells, once engrafted as alveolar epithelial cells, could limit the deleterious effects of BLM by restoring the pool of alveolar epithelial progenitor cells for the resolution of disrupted alveolar surfaces. Delivery of AEC-II-iPL cells during the established fibrotic phase, by analogy, when most patients are diagnosed with IPF, also showed beneficial effects on fibrosis. Importantly, MSC transplanted during the fibrotic phase were not able to restore the alveolar epithelium barrier or reduce fibrosis, suggesting that cells capable of directly contributing to tissue mass, such as the AEC-II-iPL cells are preferred over paracrine signaling of MSC. Our findings are in keeping with recent studies showing the efficacy of freshly isolated AEC-II cells in ameliorating fibrosis.^35,36^ Although the mechanisms of benefit have not been evaluated, our results raise the possibility that the observed therapeutic effect is at least in part due to localization of AEC-II-iPL cells within the alveolar milieu and due to their subsequent differentiation to restore damaged alveolar epithelium through the dual mechanism of engraftment and likely through paracrine or autocrine effects. Future studies, such as evaluation of cytokine antagonists that can disrupt fibrosis signaling pathways at cell engraftment sites and AEC-II-iPL cell-secreted chemokine screens for fibroblast proliferation-inhibiting factors or factors promoting collagen degradation^35^, are needed to elucidate the underlying mechanisms whereby AEC-II-iPL cells promote the recovery of injured alveolar epithelium.

Although the BLM model is not an optimal model for human IPF, it is very commonly used, and thus our results can be compared against a large body of experimental data. One of the limitations of the BLM injury model is the spontaneous repair following the initial development of fibrosis, in contrast to the progressive pathological remodeling seen in human IPF. In our BLM injury study in male mice, no significant changes of pulmonary function were detected at day 28 compared to that of saline control. We therefore assessed lung mechanics at day 21. This resulted in a shortened in situ period for cells delivered to male recipients at the fibrotic phase (male recipients: BLM^day14^ + iPL cells, sacrificed at day 21 vs. female recipients: BLM^day14^ + iPL cells, sacrificed at day 28). We speculate that the reduced efficacy observed in male recipients may be due to both the increased severity of fibrosis in male lungs as well as the shorter in situ period of the delivered cells.

Preclinical studies by Serrano-Mollar et al.^36^ demonstrated the therapeutic potential of transplantation of AEC-II cells in treating human IPF. However, the usage of freshly isolated AEC-II cells is limited by donor lung availability. Although our current data were generated from transgenic mice as a proof of principle, our technique may provide an alternative source of AEC-II cells that could be applied in human cells using non-integrative, non-viral methods of reprogramming.^65,66^ The iPL cell induction process can be optimized to obtain maximum expansion and scale-up of any cell. This may require precise regulation of expression of individual reprogramming factors to widen the time window of interrupted reprogramming, or optimization of exogenous growth factors, and/or changes to culture conditions.

Combined with other rapidly developing areas of genome editing, addressing issues such as safety of cell therapy and tolerance of allogeneic cells, the era of “designer cells” is just beginning. Our method of creating PL cells for in vitro expansion and their ability to integrate and functionally contribute to the repair of the diseased lung could be a significant component of an efficient cell-based therapy for this vital organ.

## Methods

### Animal husbandry

ROSA26-rtTA and Col1a1: tetO-4F2A mice (Jackson Labs, Cat#011004) were used to generate inducible lung epithelial cells. For in vivo studies, ROSA26-rtTA/Col1a1:: tetO-4F2A double transgenic mice were bred to actin GFP mice. There were no significant differences between heterozygotes and homozygotes with respect to inductive factor gene expression in Dox-treated AEC-II cells. Adult C57BL/6 female (6–8-week old) and male (4–5-month old) mice were used for AEC-II-iPL cell replacement therapy studies. Animals were maintained as an in-house breeding colony under specific pathogen-free conditions. All animal care protocols and procedures were performed in accordance with relevant guidelines and with approval by the Institutional Animal Care and Use Committee of the University Health Network (Toronto, Ontario, Canada).

### BLM administration and cell delivery

For BLM injury, BLM 2.5 U/kg body weight was administered intratracheally. Donor AEC-II-iPL cells or MSCs (10^6^ cells in 50 μl phosphate-buffered saline (PBS)) were delivered intratracheally 7 or 14 days after injury. Control animals received the same volume of PBS without any cells. The mice receiving cells were rotated to ensure equal dispersion of the cell suspension to both lungs.

### Measurement of respiratory mechanics

C57BL/6 male mice (4–5-month old) were anesthetized with ketamine (125 mg/kg, intraperitoneally (ip)) and xylazine (5 mg/kg, ip) and paralyzed with rocuronium (5 mg/kg, ip). Animals were tracheostomized with a blunt 18 G metal cannula, and supplementary anesthesia was employed when necessary. Conventional mechanical ventilation was maintained with a small animal ventilator (FlexiVent, SCIREQ Inc., Canada) using a tidal volume of 10 mL/kg, a frequency of 150 breaths per minute, and a positive end-expiratory pressure set at 3 cm H_2_O. PV loops were obtained with a quasi-static pressure-controlled PV ramp.

### Isolation of AEC-II cell from the mouse lung

AEC-II cells were isolated using a previously described protocol^67^ with modifications. Before lung digestion, mice were injected ip with heparin (250U/mouse) and euthanized by CO_2_ narcosis. Lungs were flushed through the right ventricle with cold PBS. Endobronchial lavage was then performed to remove alveolar leukocytes. Mouse lung was filled with porcine elastase 10 U/mL and incubated for 20 min at 37 °C. Trachea and bronchi were cut away. The remaining tissues were finely minced and incubated in 100 μg/mL of DNAse I in Dulbecco’s modified Eagle’s medium (DMEM) containing antibiotics for 10 min. The suspension was mixed with fetal bovine serum (FBS) and sieved through 100, 40, and 20 µm nylon meshes. Cells were centrifuged at 32 × *g* for 12 min and then re-suspended in red blood cell lysis buffer for 3 min, and the lysis was stopped by addition of an equal volume of PBS. Cells were centrifuged at 100 × *g* for 12 min and then re-suspended in 0.5% vol/vol FBS–PBS for all subsequent procedures.

### AEC-II cell culture

Epithelial-specific medium comprised of DMEM/F12 (Invitrogen) supplemented with 10% FBS, penicillin/streptomycin, 10 mg/mL insulin, 5 mg/mL transferrin-selenium (Sigma), epidermal growth factor (20 ng/mL; Sigma), fibroblast growth factor-10 (50 ng/mL; R&D Systems), and hepatocyte growth factor (30 ng/mL; R&D Systems).

### Isolation of MSCs from mouse bone marrow

MSCs were harvested from mouse bone marrow as previously described.^27^ Briefly, bone marrow was harvested aseptically by flushing the femurs and tibiae of donor mice with cold DMEM supplemented with 10% FBS and 1% penicillin–streptomycin using a 25G needle. BMCs were cultured for 7–10 days before MSC purification by fluorescence-activated cell sorting. MSCs were purified using antibodies against CD34, CD45, and CD11b. Sorted cells were cultured for 1–2 passages and used for in vivo delivery.

### Fluorescence-activated cell sorting and analysis

For purification of epithelial cells, fresh isolated cells were suspended and incubated in 0.5% vol/vol FBS–PBS containing an optimally pre-titered mixture of antibodies [anti-CD45, anti-CD31 (BD Biosciences), anti-EpCAM (Abcam), and relevant isotype controls] for approximately 30 min on ice. Labeled cells were washed and re-suspended at 3–5 × 10^6^ cells/mL in 0.5% vol/vol FBS–PBS. Cell viability was accessed by propidium iodide (1 μg/mL) staining. For intracellular antigen analysis, cells were fixed and stained using a Fix and Perm kit (Invitrogen) as per the manufacturer instructions. Sorting was performed using a MoFlo BRU cell sorter (Becton Dickinson), acquisition was performed using a BD LSRII analyzer (Becton Dickinson), and data were analyzed using the FlowJo software.

### Matrigel-based iPL cell induction

Feeders (MEFs) were seeded on 0.1% gelatin-coated 24-well transwell filter inserts (Corning) 1 day prior to the addition of epithelial cells. Sorted epithelial cells resuspended in 100 μL of Matrigel (BD Biosciences) prediluted 1:1 (vol/vol) with epithelial-specific (EpiS) media were added to a MEF-coated 24-well transwell filter inserts in a 24-well tissue culture plate containing 500 μL of epithelial media. To study the clonogenic capacity of passaged AEC-II cells, AEC-II cells were cultured in EpiS media for 2 weeks, passaged, and then grown in ES medium containing 1.5 μg/mL Dox (Sigma). Media was replenished three times per week. For bulk passaging, whole cultures were dissociated in Collagenase (1 mg/mL; Sigma)/Dispase (3 mg/mL; BD Biosciences) in PBS to generate a single-cell suspension.

### Immunofluorescence

Samples were fixed with 4% paraformaldehyde for 30 min and blocked with 5% goat serum and 2% bovine serum albumin (BSA) in PBS containing 0.5% Triton X-100 for 1 h. Primary antibodies were diluted in BSA/PBS, applied to samples, and incubated overnight at 4 °C. Secondary antibodies AlexaFluors 488, 532, 546, 633, or 647 (Invitrogen) were applied according to the species in which the primary antibody was used for 2 h at room temperature. Nuclear staining was performed using 2 mg/mL 4,6-diamidino-2-phenylindole (DAPI; Sigma). Stained samples were mounted with immunofluorescent mounting medium (DAKO). Appropriate non-specific IgG isotypes were used as controls. Immunoreactivities of antigens were visualized as single optical planes using an Olympus Fluoview confocal microscope and analyzed using the FV10-ASW 2.0 Viewer software.

### Real-time PCR analysis

Total RNA was prepared using the RNeasy Kit (Qiagen) as per the manufacturer’s instructions. cDNA was prepared and assayed using Superscript III (Sigma) according to the manufacturer’s protocol. Differential gene expression was determined using SYBR green detection (Roche). Real-time PCR reactions were done in triplicate for each sample. GAPDH was used as a housekeeping gene to normalize gene expression levels using the LightCycler 480 software (Roche). Normalized mRNA levels are shown as relative to the control samples (day 0 fresh isolated cells or adult lung).

### Cell retention rate measurement

Genomic DNA was isolated with the DNeasy Blood and Tissue Kit (Qiagen, Valencia, CA) and used for PCR. To quantify GFP^+^ cells retained in the recipient lungs, excised wild-type lungs were spiked with 10^4^–10^8^ GFP^+^ cells and a standard curve was generated by genomic GFP expression measured by PCR. The number of delivered GFP^+^ cells in the recipient lungs was calculated by comparing the cross-point threshold amplification value to the standard curve. Cell retention rate was calculated as the percentage of the number of cells injected at day 0.

### Microarray and data analysis

Total RNA was extracted using the RNeasy Kit (Qiagen, Canada). Equal amounts of RNA from three separate samples in each group were used for microarray. Microarray expression profiling using Mouse Gene 2.0 ST chips was performed by The Centre for Applied Genomics (The Hospital for Sick Children, Toronto, Canada).

### Statistics

Statistical analysis was performed using the GraphPad Prism 5.0 statistical software (San Diego, CA, USA). The statistical significance of multiple groups was compared to each other using Tukey’s multiple comparison test analysis of variance. A *p* value of <0.05 was considered significant.

## Electronic supplementary material

Supplementary information

## Data Availability

The microarray data were deposited in the Gene Expression Omnibus under accession number GEO: GSE115247. The data sets generated during this study are available from the corresponding authors on request.
