# Supplementary material for: Interrupted reprogramming of alveolar type II cells induces progenitor-like cells that ameliorate pulmonary fibrosis
Source: NPJ Regen Med. 2018 Sep 4;3:14. doi: 10.1038/s41536-018-0052-5 (PMC6123410; doi:10.1038/s41536-018-0052-5)
Supplement: Supplementary file 1 — Supplementary information [file 41536_2018_52_MOESM1_ESM.pdf]

Supplementary figure and text

Figure S1

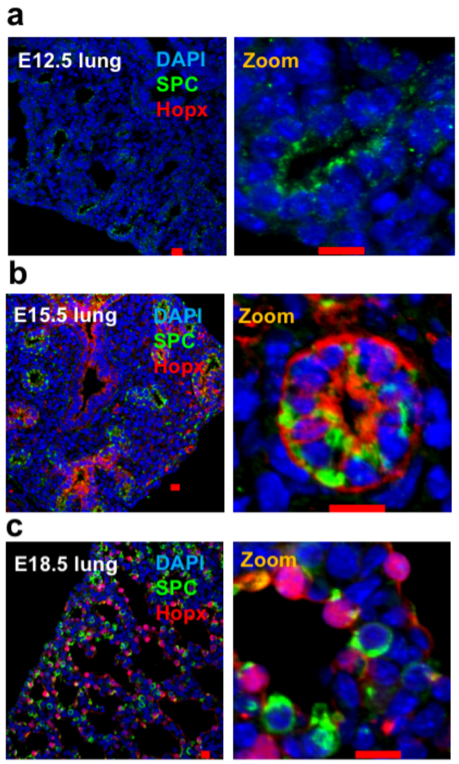

Figure S1. Time course of expression of Hopx and SPC in embryonic lungs

Confocal microscopy images depicting the expression of Hopx and SPC in (a) E12.5, (b) E15.5 and (c) E18.5 lungs, with nuclear stain DAPI (blue), SPC (green) and Hopx (red). Scale bar, 10μm.

Figure S2

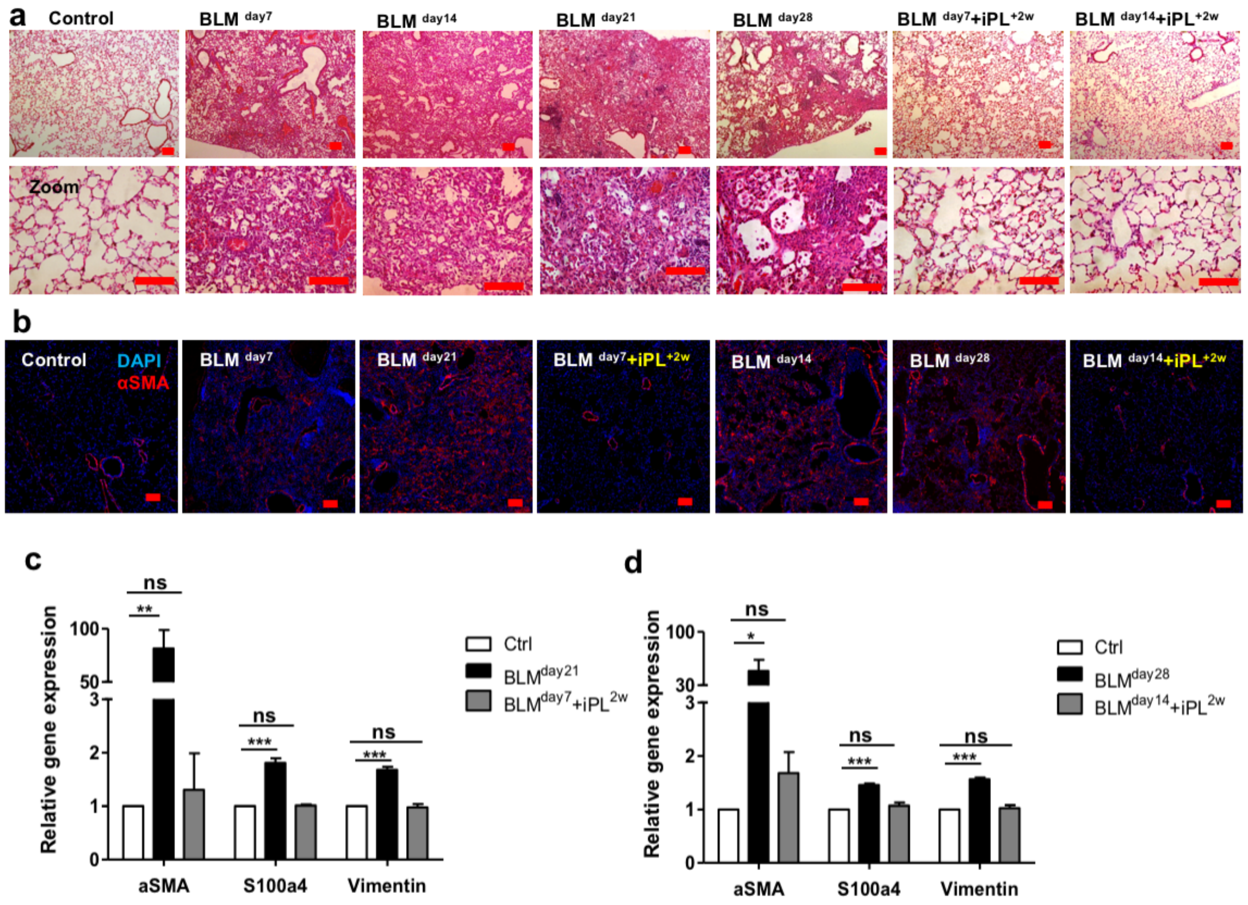

Figure S2. Transplant of AEC-II-iPL cells ameliorates bleomycin-mediated pulmonary fibrosis

(a) Representative images of hematoxylin–eosin stained lung sections in all the experimental groups after 7, 14, 21 and 28 days of BLM-induced pulmonary fibrosis. (b) Immunostaining of lung sections in all experimental groups with nuclear stain DAPI (blue) and αSMA (red). Expression of mesenchymal genes (αSMA, S100a4 and vimentin) in BLM non-treated (c, BLM^{day21}) (d, BLM^{day28}) and BLM-treated (c, $BLM^{day7}+iPL^{+2w}$) (d, $BLM^{day14}+iPL^{+2w}$) recipient lungs, as measured by qRT-PCR

comparing fold-differences in expression to saline treated control (Ctrl) lung. For c and d, values are mean ± S.D. of three independent biological replicates. *, $p<0.05$; **, $p<0.001$; ***, $p<0.0001$. Scale bar, 100 μm.

Tables

Table 1: Tabulated list of all qPCR primers used in the studies.

qPCR primers			
**Gene name**	**Primer sequence 5'-3'**		**length**
mCol4F2A	Forward	GGCTGGAGATGTTGAGAGCAA	21
	Reverse	AAAGGAAATCCAGTGGCGC	19
Sftpc	Forward	GCAAAGAGGTCCTGATGGAG	20
	Reverse	GCAGTAGGTTCCTGGAGCTG	20
AQP5	Forward	ATGAACCCAGCCCGATCTTT	20
	Reverse	ACGATCGGTCCTACCCAGAAG	21
α-SMA	Forward	CCCAGACATCAGGGAGTAATGG	22
	Reverse	TCTATCGGATACTTCAGCGTCA	22
S100A4	Forward	TCCACAAATACTCAGGCAAAGAG	23
	Reverse	GCAGCTCCCTGGTCAGTAG	19
Vimentin	Forward	CGTCCACACGCACCTACAG	19
	Reverse	GGGGGATGAGGAATAGAGGCT	21

Table 2: Tabulated list of all antibodies used in the studies

Antibody name	Company	Cat. #	Clone name	Lot #	Validation
CD45	BD Pharmingen	553081	30-F11	3221754	Adult lung cells
CD31	BD Pharmingen	553373	MEC 13.3	2286929	Adult lung cells
EpCAM	Abcam	ab95641	G 8.8	GR267522-3	Adult lung cells
SPC	Millipore	AB3786		2464523	Adult lung tissue
	Santa Cruz	sc-7706	M-20	E1215	Adult lung tissue
Hopx	Santa Cruz	sc-398703	E-1	H0714	Adult lung tissue
a-SMA	Abcam	Ab7817	1A4	GR119216-4	Adult lung tissue
Integrin α6	Biolegend	313611	GoH3	B143404	Adult lung cells
Integrin β4	Abcam	ab182120	EPR17517	GR214899-1	Adult lung tissue
	Biolegend	123608	346-11A	B144521	Adult lung cells
CD74	BD Pharmingen	561941	In-1	313611	Adult lung cells
LAMP3	Novusbio	DDX0192P-100	1006F7.05	DDX0192-011	Adult lung tissue

Nanog	Abcam	ab80892		GR40243-10	R1-ESC cells
	BD Pharmingen	560259	M55-312	9933	R1-ESC cells
GFP	Invitrogen	A-21311		1567217	Adult lung tissue
AQP5	Abcam	ab104751		GR34237-29	Adult lung tissue
SSEA-1	Santa Cruz	sc-21702	480	E0710	R1-ESC cells
E-cadherin	Abcam	ab11512		GR127224-1	Adult lung cells
